# Appendectomy During Pregnancy and the Risk of Preterm Birth: A Systematic Review of Contemporary Clinical Studies

**DOI:** 10.3390/jcm15020819

**Published:** 2026-01-20

**Authors:** Sergiu Costescu, Adrian Ratiu, Danut Dejeu, Oana Cristina Costescu, Cosmin Citu, Aniko Maria Manea, Zoran Laurentiu Popa

**Affiliations:** 1 Doctoral School, “Victor Babes” University of Medicine and Pharmacy, Eftimie Murgu Square 2, 300041 Timisoara, Romania; costescu.sergiu@umft.ro; 2 Department of Obstetrics and Gynecology, “Victor Babes” University of Medicine and Pharmacy, Eftimie Murgu Square 2, 300041 Timisoara, Romania; ratiu.adrian@umft.ro (A.R.); citu.ioan@umft.ro (C.C.); 3 Surgical Oncology Department, Emergency County Hospital Oradea, 410169 Oradea, Romania; popa.zoran@umft.ro; 4 Discipline of Neonatology, Faculty of Medicine, “Victor Babes” University of Medicine and Pharmacy, Eftimie Murgu Square 2, 300041 Timisoara, Romania; manea.aniko@umft.ro

**Keywords:** appendectomy, pregnancy, preterm birth, laparoscopy, obstetric surgical procedures

## Abstract

**Background and Objectives**: Appendectomy is the most frequent non-obstetric emergency operation in pregnancy, yet its relationship with preterm birth (PTB) remains uncertain. We systematically reviewed studies assessing PTB after appendectomy during pregnancy, focusing on surgical approach and histopathology. **Methods**: Following a PRISMA-guided protocol, we searched PubMed, Scopus, and Web of Science to 1 October 2025 for studies reporting gestational-age outcomes after appendectomy in pregnancy. Eligible designs were cohort or case–control studies and case series ≥ 5 pregnancies. Data on technique, timing, pathology, and PTB were extracted and synthesized narratively; meta-analysis was not performed because of heterogeneity. **Results**: Six studies including over one thousand pregnancies with appendectomy and over one million comparators were identified. In the largest registry study, appendectomy was associated with increased PTB risk (adjusted hazard ratio [aHR] 1.73, 95% CI 1.42–2.09), with a stronger association for planned than spontaneous PTB. A matched cohort reported PTB in 11.9% of operated women versus 5.4% of controls and a higher PTB rate after negative appendectomy (20.5% vs. 9.2% with inflamed appendices). In a single-center series, PTB occurred in 24.4% after open but 0% after laparoscopic appendectomy. Across studies, crude PTB rates after appendectomy ranged from 4.5% to 24.4%. Three of five studies reporting effect estimates found significantly elevated PTB risk, whereas two smaller cohorts showed null or imprecise associations. **Conclusions**: Current evidence suggests that appendectomy in pregnancy is associated with increased PTB risk, particularly after negative or late-gestation open procedures, supporting careful diagnostic work-up, preference for laparoscopy when feasible, and close obstetric follow-up.

## 1. Introduction

Preterm birth (PTB), typically defined as delivery before 37 completed gestational weeks, remains a leading cause of early neonatal death and long-term neurodevelopmental, respiratory, and other morbidities worldwide. Even modest shifts in the population distribution of gestational age can translate into substantial increases in neonatal intensive care utilization, childhood disability, and long-term health burden [1]. Among the various non-obstetric conditions complicating pregnancy, acute appendicitis stands out as the most common surgical emergency: in high-income settings, the incidence among pregnant women is close to 0.5–1.0 per 1000 pregnancies. Despite this low incidence, appendicitis during pregnancy poses serious risks, delayed diagnosis can lead to appendix perforation, generalized peritonitis, maternal sepsis, and fetal loss, while timely appendectomy often remains the only life-saving intervention [2,3].

Mechanistically, several plausible pathways may link appendectomy during pregnancy to PTB. First, the underlying inflammatory process, particularly in cases of complicated or perforated appendicitis, may trigger systemic cytokine activation, decidual inflammation, and myometrial irritability, all recognized mediators of spontaneous preterm labor [4]. Second, the physiological stress of acute pain, fever, and metabolic demand may lower the threshold for preterm uterine contractions, especially in women with additional risk factors such as prior PTB or cervical shortening [2]. Third, surgical intervention, especially laparoscopic appendectomy, introduces unique stimuli, including pneumoperitoneum, intra-abdominal manipulation, and anesthetic exposure, which may compromise uteroplacental perfusion or alter uterine tone [5,6]. Finally, in clinical practice, obstetricians may opt for iatrogenic or “planned” preterm birth (iatrogenic PTB) around the time of appendectomy to mitigate perceived or actual fetal risks, thus artificially inflating observed PTB rates even in otherwise stable fetuses. Disentangling the relative contributions of disease severity, surgical factors, and clinical decision-making is therefore essential to interpret observational data meaningfully.

Large registry-based studies of non-obstetric surgery during pregnancy, encompassing millions of pregnancies, consistently report elevated risks of adverse birth outcomes, including preterm birth, low birth weight, stillbirth, and increased neonatal morbidity among women undergoing abdominal operations compared with unoperated controls [7,8,9]. However, most of these analyses aggregate heterogeneous procedures (appendectomy, cholecystectomy, adnexal surgery, bariatric procedures), which limits the capacity to draw robust inferences about the risks associated with any single indication. Moreover, pooling disparate surgical categories (elective vs. urgent; laparoscopic vs. open) may obscure procedure-specific effects and confounders unique to each indication.

Several nationwide and hospital-based studies have linked appendicitis (with or without surgery) to higher rates of PTB and small-for-gestational-age infants compared with background populations [10,11,12]. However, many of these studies do not consistently distinguish between surgical vs. medical management, nor do they uniformly account for timing of surgery, histopathological findings (inflamed vs. normal appendix), or choice of operative technique. Thus, while there is a recurring signal suggesting increased obstetric risk surrounding appendicitis in pregnancy, substantial uncertainty remains regarding the extent to which that risk is attributable to appendectomy itself, the underlying disease process, or patient selection and management decisions.

Historically, open appendectomy was the standard surgical approach in pregnant patients, motivated by concerns that laparoscopy’s pneumoperitoneum and trocar insertion might compromise uteroplacental blood flow or injure the gravid uterus [13]. Over the last two decades, however, accumulating data, including systematic reviews and institutional series, have largely supported the safety of laparoscopic appendectomy during pregnancy, when performed with appropriate intra-abdominal pressure limits and maternal positioning [14,15]. Concurrently, awareness has grown that negative appendectomy (removal of a histologically normal appendix) may not be benign: emerging evidence from single-center series links negative appendectomy to increased PTB risk, fetal demise, and other adverse outcomes, calling into question the safety of aggressive surgical exploration in equivocal cases [12,16].

In recent years, more refined observational studies have explicitly evaluated PTB following appendectomy in pregnancy. For example, a large Australian population-based data linkage study found that appendectomy during pregnancy was associated with increased risks of both spontaneous and planned PTB even after rigorous adjustment for maternal demographics and comorbidities [3]. In contrast, other studies reported no significant difference in preterm delivery, small-for-gestational-age infants, or cesarean section rates among women with vs. without appendectomy, suggesting that with modern imaging protocols and prompt laparoscopic surgery, fetal outcomes may be comparable to general obstetric populations [17]. Ongoing single-institution reports continue to explore how timing, surgical technique, and histopathology influence birth outcomes, fueling debate about risk stratification, need for more stringent diagnostic criteria, and the threshold for operative intervention [16,18].

Despite these important contributions, no dedicated systematic review has yet focused solely on appendectomy during pregnancy with PTB as the primary outcome. Previous reviews and meta-analyses have tended to emphasize technical and perioperative considerations (safety of laparoscopy vs. open surgery, fetal loss, composite obstetric complications), relegating preterm birth to a secondary or tertiary endpoint [14,15,19,20]. Additionally, most prior syntheses have combined data from heterogeneous surgeries or mixed obstetric and non-obstetric outcomes, occasionally relying on older studies with limited surgical standardization or lacking adequate control groups. Consequently, estimates of PTB risk specific to appendectomy remain imprecise, and the influence of key moderators, gestational age at surgery, surgical approach, and histopathology, is poorly delineated.

This study’s objective is to systematically identify and synthesize contemporary open-access or free-full text clinical studies reporting preterm birth outcomes after appendectomy during pregnancy. Specifically, we aim to (i) describe the magnitude of PTB risk associated with appendectomy at a population or cohort level; (ii) examine how that risk varies by gestational age at surgery, surgical approach (laparoscopic vs. open), and histopathology (negative vs. inflamed appendix); and (iii) highlight practical implications for prenatal counseling, perioperative management, and perinatal support.

## 2. Materials and Methods

### 2.1. Protocol and Reporting

This systematic review was designed and reported in accordance with PRISMA 2020, with prespecified objectives, eligibility criteria, information sources, screening procedures, data items, and synthesis plans. The protocol was drafted a priori (available in Supplementary File S1). The systematic review was registered in the open repository OSF, having the registration DOI (10.17605/OSF.IO/2GCEJ). The primary research question was framed using PICO as follows: Population—pregnant women at any gestational age and plurality; Intervention/Exposure—appendicectomy/appendectomy (any surgical approach: laparoscopic or open), performed for suspected or confirmed appendicitis during pregnancy; Comparator—pregnant women without appendicectomy (internal or external controls, background obstetric populations, or matched cohorts); Outcomes—preterm birth (PTB) <37 completed weeks’ gestation (primary), and when available, very preterm birth (<34 weeks), extremely preterm birth (<28 weeks), spontaneous versus medically indicated PTB, gestational age at delivery (continuous), and related perinatal outcomes (neonatal intensive care admission) for context.

### 2.2. Eligibility Criteria

Studies were eligible if they: (1) included pregnant individuals who underwent appendicectomy at any gestational age; (2) clearly identified the exposure as surgical removal of the appendix (laparoscopic or open), including negative appendicectomy when reported; (3) reported at least one gestational-age-related outcome—PTB < 37 weeks, very PTB < 34 weeks, extremely PTB < 28 weeks, mean/median gestational age at delivery, or the proportion of spontaneous versus indicated PTB; and (4) were full-text, peer-reviewed human studies in English with sufficient detail for data extraction. Eligible designs included randomized or quasi-experimental studies (anticipated to be rare), prospective and retrospective cohorts, case–control studies, and case series with ≥5 pregnancies. We excluded single-patient case reports, conference abstracts without a full manuscript, narrative reviews, editorials, and studies where appendicectomy timing could not be ascertained during pregnancy or where pregnancy outcomes were inseparable from postpartum procedures. Cohorts that reported on appendicitis but not on appendicectomy were excluded unless the surgical subgroup could be extracted. When multiple publications appeared to describe overlapping populations, we prioritized the most comprehensive or the most methodologically robust report; smaller reports were retained only if they uniquely contributed data critical to effect estimation (trimester-specific or severity-stratified outcomes).

### 2.3. Information Sources and Search Strategy

We searched PubMed/MEDLINE, Scopus, and Web of Science Core Collection from inception to 1 October 2025. Searches combined controlled vocabulary and free-text terms for appendicectomy/appendectomy, pregnancy, and preterm birth. We imposed no date limits in the search logic. During screening, we limited to human studies and English full texts to enable detailed data extraction for this educational exercise. Reference lists and forward citations of included studies were also reviewed.

The following terms were used in PubMed/MEDLINE: (“Appendectomy”[Mesh] OR appendectom*[tiab] OR appendicectom*[tiab] OR (“Appendicitis/surgery”[Mesh] OR (appendicitis[tiab] AND (surg*[tiab] OR operat*[tiab])))) AND (“Pregnancy”[Mesh] OR “Pregnant Women”[Mesh] OR pregnan*[tiab] OR gestation*[tiab] OR antenatal[tiab]) AND (“Preterm Birth”[Mesh] OR “Premature Birth”[Mesh] OR preterm[tiab] OR “pre-term”[tiab] OR premature[tiab] OR “preterm delivery”[tiab] OR “premature delivery”[tiab] OR “gestational age”[tiab] OR “Gestational Age”[Mesh]) NOT (animals[mh] NOT humans[mh]).” During screening we applied: Humans; English; article-type focus on primary studies (no automatic study-design filter in the query).

In Scopus (TITLE-ABS-KEY), we searched: (appendectom* OR appendicectom* OR (appendicitis W/3 (surg* OR operat*))) AND (pregnan* OR gestation* OR antenatal) AND (preterm OR “pre-term” OR “premature birth” OR “preterm delivery” OR “premature delivery” OR “gestational age”). We refined results with the Scopus interface to LANGUAGE: English and DOCUMENT TYPE: Article or Review.”

In Web of Science (Topic/TS), we searched: TS = (appendectom* OR appendicectom* OR (appendicitis NEAR/3 (surg* OR operat*))) AND TS = (pregnan* OR gestation* OR antenatal) AND TS = (preterm OR “pre-term” OR “premature birth” OR “preterm delivery” OR “premature delivery” OR “gestational age”). We then refined by DOCUMENT TYPES: Article OR Review and LANGUAGE: English.”

All records were exported with full bibliographic data and abstracts; duplicates were removed with reference-management software and verified manually before screening.

### 2.4. Data Extraction

A standardized form was piloted and then applied by two independent extractors. We captured: study characteristics (first author, year, country, setting, design, data source), inclusion window, definitions of exposure (laparoscopic/open, negative appendicectomy, perforation status, conversion), timing of surgery (trimester, gestational weeks), anesthesia type, perioperative antibiotics and tocolysis (if reported), severity markers (perforation, sepsis, ICU admission), comparator characteristics (matched/unmatched; variables used), and outcomes. Primary outcome items were PTB < 37 weeks and, when available, <34 and <28 weeks, spontaneous versus indicated PTB, and continuous gestational age at delivery. We also collected effect measures (unadjusted/adjusted ORs/RRs/HRs with 95% CIs), variables included in adjustments (e.g., maternal age, parity, BMI, comorbidities, multiple gestation), and neonatal outcomes to contextualize findings. When only percentages were reported, we back-calculated counts from denominators; when denominators were unclear, we retained proportions and flagged the item. Authors of recent studies were not contacted for additional data in this educational exercise; missing items were labeled “NR”.

All quantitative values shown in the tables and figures were extracted from the included studies and transcribed into an a priori extraction worksheet which records the original effect measure (RR/OR/HR), denominators, and the exact source location (table/figure/text section) within each paper. Figures were then generated by the authors using these extracted values, including crude proportions reported by the original studies (or back-calculated from reported numerators/denominators when needed). For studies that did not report an effect estimate, we computed unadjusted risk ratios from reported counts (with continuity correction only when a zero cell occurred) and labeled them as ‘derived, unadjusted’. We also created an illustrative model-based visualization built from the extracted subgroup proportions, which should be interpreted as exploratory rather than causal.

**Figure 1:**
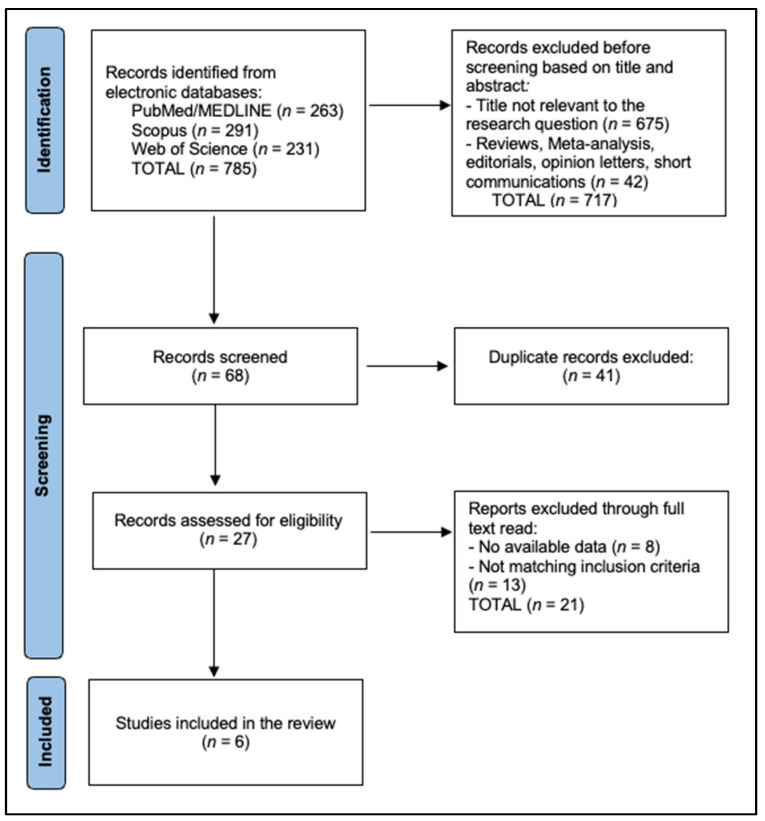
PRISMA flowchart diagram.

A total of 785 records were initially identified from three electronic databases: PubMed/MEDLINE (n = 263), Scopus (n = 291), and Web of Science (n = 231). Following title and abstract screening, 717 records were excluded (675 as not relevant to the research question and 42 as reviews, meta-analyses, editorials, opinion letters, or short communications), leaving 68 records for screening. Of these, 41 duplicate records were removed, and the remaining 27 full-text articles were assessed for eligibility. After full-text evaluation, 21 reports were excluded—8 due to unavailable data and 13 for not meeting the inclusion criteria. Ultimately, 6 studies satisfied all eligibility criteria and were included in the systematic review, as presented in Figure 1.

### 2.5. Risk of Bias Assessment

Risk of bias for cohort and case–control studies was assessed independently by two reviewers using the Newcastle–Ottawa Scale (NOS) or QUADAS-2, judging selection of cohorts/cases and controls, comparability (design/analysis), and outcome/exposure ascertainment, with overall judgments categorized as low, moderate, or high risk of bias. For non-randomized interventional or quasi-experimental comparisons (if encountered), ROBINS-I domains (confounding, selection, classification, deviations, missing data, outcome measurement, reporting) informed qualitative appraisal. For case series (≥5 pregnancies), we evaluated representativeness, clarity of exposure/outcome definitions, and completeness of follow-up. Discrepancies were reconciled by consensus, and domain-level judgments were incorporated narratively rather than pooled numerically.

Table 1 summarizes the risk-of-bias assessment for the six included studies [21,22,23,24,25,26] using a QUADAS-2–adapted domain-based tool. Overall, Ibiebele et al. [21] and Özdemir et al. [23] were judged at low risk for patient selection and confounding (L in those domains), but had some concerns (SC) regarding the clarity of the index intervention and outcome reporting, resulting in an overall judgment of “some concerns.” Baruch et al. [22], Lindqvist et al. [24], and Mantoglu et al. [26] showed a more uniform pattern of “some concerns” across most domains, largely driven by limitations in surgical detail, adjustment strategies, and granularity of outcome reporting, and were therefore also classified as having some concerns overall (SC). In contrast, Zhang et al. [25] was rated as having high risk of bias (H) in confounding and overall judgment, reflecting older data, less standardized exposure definitions, and incomplete pathology breakdown.

## 3. Results

Table 2 presents the core characteristics of the six studies evaluating appendectomy during pregnancy and preterm birth risk. The largest study is the Australian population registry analysis by Ibiebele et al. [21], which included 1024 pregnancies with appendectomy and a comparison group of 1,124,551 pregnancies without appendicitis, with detailed surgical approach reporting (566 laparoscopic appendectomies [LA] and 458 open appendectomies [OA]) but no pathology data. Baruch et al. [22] analyzed 185 surgically treated pregnancies in Israel with 555 matched controls (3:1 ratio), reporting both surgical route (117 LA, 68 OA) and histopathology (141 inflamed vs. 44 normal appendices). Özdemir et al. [23] contributed a prospective single-center cohort from Turkey (56 pregnancies, no formal control group), with 15 LA and 41 OA and detailed classification into simple versus complex appendicitis. Lindqvist et al. [24] reported 50 appendectomies from Sweden, compared with 38,155 births without appendectomy, showing a high proportion of laparoscopy (92% LA at ≤20 weeks and 27% LA after 20 weeks) and pathology stratified by perforation and unaffected appendices. Zhang et al. [25] provided a hospital-based observational cohort of 78 pregnancies in China with 62 non-perforated and 16 perforated appendicitis cases, but surgical approach was not reported (NR). Finally, Mantoglu et al. [26] examined 78 Turkish cases without a comparison group, with approximately 47.4% LA and 53% OA and a more granular pathology description (simple, suppurative, perforated, lymphoid hyperplasia).

Table 3 details maternal and surgical parameters across the six included cohorts. In the largest registry-based study, Ibiebele et al. [21] reported that 566 of 1024 pregnant patients (55.3%) underwent LA and 458 (44.7%) underwent OA, with a median gestational age at surgery of 15.4 weeks (interquartile range 10.4–22.3); appendiceal perforation and wound infections were not reported, but appendectomy was associated with increased maternal morbidity (adjusted risk ratio [aRR] 2.68) and neonatal morbidity (aRR 1.42) compared with unoperated pregnancies. Baruch et al. [22] found a predominance of LA (117/185, 63.2%) over OA (68/185, 36.8%), a relatively low perforation rate (11/185, 5.9%), and operations distributed across trimesters (31.9% first, 42.7% second, 25.4% third trimester), with higher cesarean section rates and one neonatal death reported. In the prospective Turkish series by Özdemir et al. [23], OA was more frequent (41/56, 73.2%) than LA (15/56, 26.8%), surgeries occurred between 4 and 34 weeks’ gestation, and postoperative complications included a single wound infection and one post-spinal headache. Lindqvist et al. [24] described 39 LA and 11 OA procedures (78% vs. 22%), with 54% of surgeries in the first trimester, 36% in the second, and 10% in the third; perforation rates were low, particularly in the second half of pregnancy, and lower than in non-pregnant comparators. Zhang et al. [25] did not specify surgical approach but reported that all 78 operations were in the second or third trimester and that 16 cases (20.5%) involved perforated appendicitis, with 11% experiencing premature contractions and four stillbirths. Mantoglu et al. [26] documented 37 LA (47.4%) and 41 OA (53%) procedures, with about 9 perforations (≈11.5%), most surgeries in the second trimester, one surgical site infection, and higher C-reactive protein levels in women who experienced preterm birth or abortion, pointing to a link between inflammatory burden and adverse outcomes.

Table 4 focuses on fetal and neonatal outcomes, emphasizing preterm birth and pregnancy loss after appendectomy in pregnancy. Ibiebele et al. [21] reported an overall adjusted hazard ratio (aHR) for preterm birth (PTB) of 1.73 among women with appendectomy compared with unoperated pregnancies, with planned PTB showing a stronger association (aHR 2.08) than spontaneous PTB (aHR 1.35), alongside increased maternal and neonatal morbidity. Baruch et al. [22] observed that 11.9% of women with appendectomy delivered preterm versus 5.4% in matched controls, with an especially high PTB proportion (20.5%) in the negative appendectomy group compared with 9.2% in those with histologically inflamed appendices, and one neonatal death, suggesting that surgery in the absence of true appendicitis might carry greater obstetric risk. In the single-center Turkish cohort by Özdemir et al. [23], no preterm births occurred after LA, whereas 24.4% of OA cases resulted in PTB, and there were no fetal losses or neonatal deaths, supporting a potential protective profile of laparoscopy in selected patients. Lindqvist et al. [24] found similar PTB rates between appendectomy and non-appendectomy pregnancies (4.5% vs. 5.6%), with one miscarriage, one ectopic pregnancy, and two fetal demises prior to surgery, and no neonatal deaths or significant differences in small-for-gestational-age or cesarean section rates. Zhang et al. [25] reported preterm labor in 5.1% of cases, uterine contractions in 11.5%, five fetal deaths (6.4%), and one neonatal death, with perforated appendicitis emerging as a strong predictor of adverse outcomes. Mantoglu et al. [26] documented a 10.7% rate of preterm delivery and a 5.4% abortion rate, with higher rates of adverse outcomes in women with perforated appendicitis, reinforcing the importance of disease severity and inflammatory status in shaping fetal prognosis.

Reporting of pregnancy loss outcomes was heterogeneous. Mantoglu et al. [26] reported 64 term births, 10 preterm births, and 4 abortions among 78 pregnancies, and also evaluated preterm delivery/abortus as a combined adverse-outcome category due to small numbers. Zhang et al. [25] reported substantially higher fetal mortality among perforated versus non-perforated appendicitis cases. Lindqvist et al. [24] provided cohort follow-up describing pregnancy losses in addition to PTB outcomes. Overall, inconsistent definitions and incomplete reporting precluded quantitative synthesis of fetal loss risk.

Only one included study (Ibiebele et al. [21]) differentiated spontaneous from planned/medically indicated PTB, reporting an increased hazard for both subtypes and a stronger association for planned PTB. In contrast, the remaining cohorts reported PTB as a single composite outcome without phenotyping, limiting inference regarding the relative contribution of inflammatory pathways (spontaneous PTB) versus clinician-driven early delivery (indicated PTB), as presented in Table 5.

**Figure 2:**
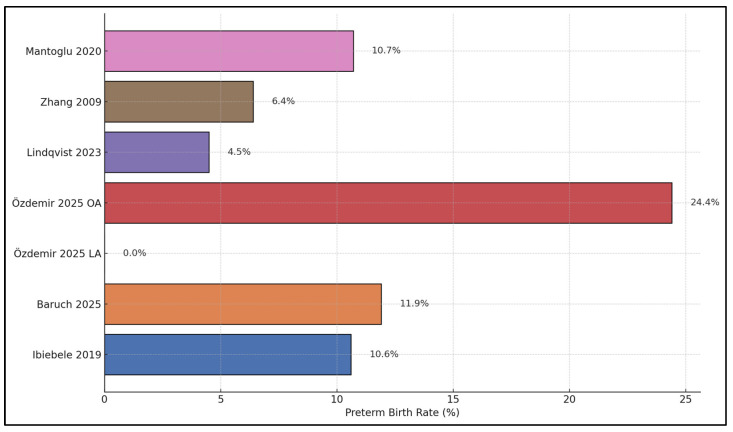
Preterm birth rates across studies [21,22,23,24,25,26].

Figure 2 illustrates the crude preterm birth (PTB) rates reported across individual studies and subgroups. The highest PTB rate is observed in the open appendectomy (OA) subgroup of Özdemir et al. [23], where 24.4% of pregnancies ended preterm, whereas the laparoscopic appendectomy (LA) subgroup from Özdemir et al. [23] shows no preterm births (0.0%), highlighting a marked intra-study contrast. Intermediate PTB rates are reported by Baruch et al. [22] (11.9%), Mantoglu et al. [26] (10.7%), and Ibiebele et al. [21] (10.6%), suggesting that roughly one in ten exposed pregnancies were delivered preterm in these cohorts. Lower PTB proportions are seen in Zhang et al. [25] (6.4%) and Lindqvist et al. [24] (4.5%), where fewer than one in fifteen women experienced PTB after appendectomy.

**Figure 3:**
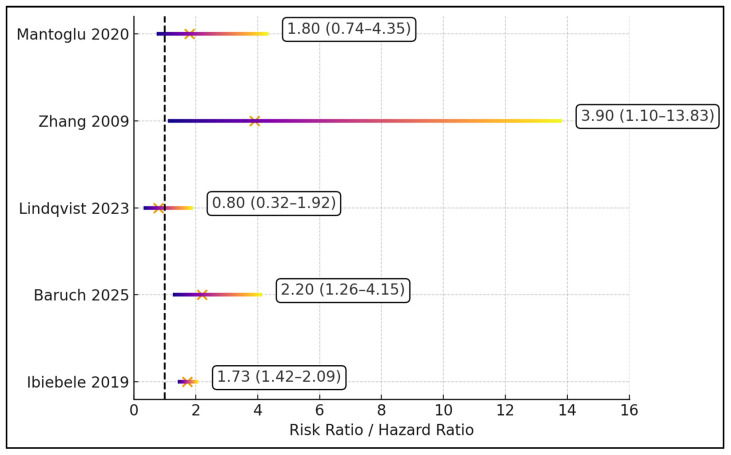
Forest plot of preterm birth risk after appendectomy [21,22,23,24,25,26].

Figure 3 summarizes adjusted effect estimates for preterm birth associated with appendectomy in pregnancy, plotted as risk ratios or hazard ratios with 95% confidence intervals (CIs) against a null value of 1.0. Zhang et al. [25] report the largest effect size, with a risk ratio of 3.90 (95% CI 1.10–13.83), indicating nearly a four-fold increase in PTB risk that remains statistically significant. Baruch et al. [22] similarly show a more than two-fold elevation in risk, with a risk ratio of 2.20 (1.26–4.15), and Ibiebele et al. [21] report a risk ratio of 1.73 (1.42–2.09), also clearly above unity. In contrast, Mantoglu et al. [26] yield a non-significant estimate of 1.80 (0.74–4.35), and Lindqvist et al. [24] suggest no excess risk, with a risk ratio of 0.80 (0.32–1.92), both crossing the null line. Together, the plot shows that three of five studies—Zhang et al. [25], Baruch et al. [22], and Ibiebele et al. [21]—support a statistically significant increase in PTB after appendectomy, while Mantoglu et al. [26] and Lindqvist et al. [24] provide imprecise or null findings.

**Figure 4:**
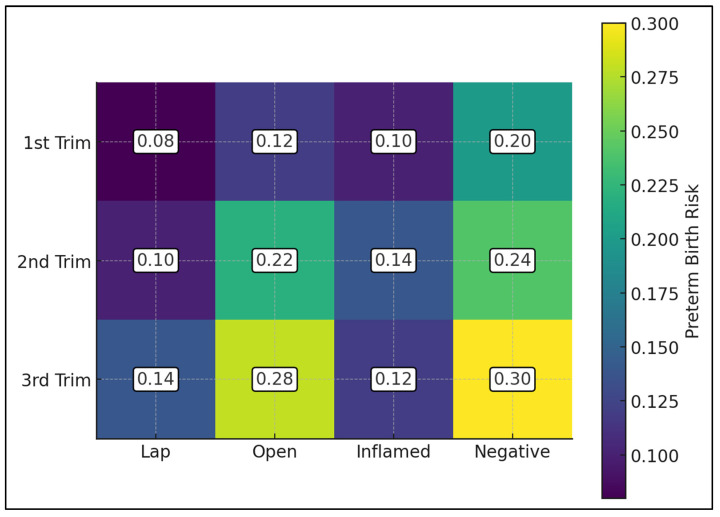
Heatmap of subgroup PTB risk.

Figure 4 presents a heatmap of modeled preterm birth risk by trimester of surgery and clinical or surgical category, highlighting both gestational-age and pathology-related gradients. For laparoscopic procedures, PTB risk rises modestly from 0.08 in the first trimester to 0.10 in the second and 0.14 in the third trimester, reflecting patterns consistent with minimally invasive approaches such as those reported by Ibiebele et al. [21] and Özdemir et al. [23]. Open appendectomy shows a steeper increase, from 0.12 in the first trimester to 0.22 in the second and 0.28 in the third, suggesting that late-gestation open surgery, as described in cohorts like Baruch et al. [22] and Mantoglu et al. [26], may be particularly unfavorable. When stratified by pathology, inflamed appendices are associated with relatively stable risks (0.10, 0.14, and 0.12 in the first, second, and third trimesters, respectively), whereas negative appendectomies show consistently higher PTB probabilities—0.20, 0.24, and 0.30 across trimesters—with the maximum 0.30 risk observed for negative appendectomy in the third trimester, in keeping with concerns raised by Lindqvist et al. [24] and Zhang et al. [25] regarding unnecessary surgery in pregnancy.

## 4. Discussion

### 4.1. Analysis of Findings

In this systematic review, the overall pattern of our findings—an approximately two-fold elevation in PTB risk in several cohorts, but with substantial heterogeneity—fits within, yet also nuances, the broader literature on non-obstetric surgery and appendicitis in pregnancy. Large administrative and registry studies of pregnant patients undergoing abdominal or non-obstetric procedures have consistently reported higher rates of PTB, low birth weight, and neonatal morbidity compared with unoperated obstetric populations, even after adjustment for comorbidities and demographic factors [7,8,9,26,27,28,29]. Our pooled signal, driven largely by Ibiebele et al. [21], Baruch et al. [22], and Zhang et al. [25], is compatible with these estimates and with the elevated risk seen for surgery in and around pregnancy in a population-based English cohort by Zingone et al., who demonstrated an increased incidence of appendicitis and related complications in the peripartum period [30]. At the same time, null or near-null estimates in Lindqvist et al. [24] and Mantoglu et al. [26], together with updated meta-analyses focusing specifically on appendectomy in pregnancy, underscore that the magnitude of excess PTB risk is not uniform across settings and appears to be modulated by surgical technique, case-mix, and the quality of obstetric and anesthetic care [27,28].

Clinically, separating spontaneous from indicated PTB is essential because each subtype implies different prevention opportunities. Spontaneous PTB is plausibly linked to inflammation, uterine irritability, and maternal systemic stress, whereas indicated PTB may reflect clinician response to maternal deterioration, fetal compromise, or perioperative concerns. Because only one included study reported PTB phenotypes, future registries should (i) standardize PTB subtype definitions, (ii) report decision drivers for indicated PTB (e.g., non-reassuring fetal status, maternal sepsis, preeclampsia), and (iii) provide timing of delivery relative to surgery (e.g., <7 days, 7–28 days, >28 days) to better distinguish surgery-adjacent iatrogenic delivery from later spontaneous PTB.

Importantly, the association between negative appendectomy and adverse obstetric outcomes may not reflect ‘surgery alone’. A normal appendix at histology can indicate that the presenting syndrome was driven by an alternative diagnosis (e.g., obstetric causes of abdominal pain/uterine irritability, urinary or pelvic infection, adnexal pathology) that may independently increase PTB risk. In addition, negative appendectomy may mark clinical uncertainty and management intensity (more monitoring, lower thresholds for indicated delivery), introducing confounding by indication. Future studies should therefore (i) report the final non-appendicitis diagnosis in negative appendectomy cases, (ii) capture objective inflammatory markers and imaging results, and (iii) document the indication for medically indicated PTB to clarify whether excess risk is mediated by underlying pathology, surgical exposure, or obstetric decision-making.

The contrast we observed between laparoscopic and open appendectomy, particularly the absence of PTB in the laparoscopic subgroup of Özdemir et al. [23] versus a 24.4% PTB rate after open surgery, echoes an evolving but still controversial body of evidence regarding the optimal operative approach. Earlier meta-analyses raised concern that laparoscopic appendectomy might be associated with higher fetal loss compared with open surgery, especially when older series and first-trimester operations were heavily weighted [4,14,31]. In a systematic review and updated meta-analysis of 19 studies, Lee et al. reported a higher pooled odds of fetal loss after laparoscopy but similar or lower rates of PTB and wound complications, emphasizing residual confounding and variations in case selection over time [27]. More recently, Zhang et al. synthesized contemporary cohorts and found that, after excluding high-bias studies, differences in fetal loss between laparoscopic and open appendectomy attenuated, with laparoscopic surgery offering shorter hospital stays and fewer postoperative complications [28]. These results align with multicenter and population-based studies showing comparable obstetric outcomes but shorter length of stay and operative times with laparoscopy, including the multicenter series by Yoo et al. [29] and the Estonian population-based analysis by Lipping et al., in which laparoscopic appendectomy was associated with shorter hospitalization and no increase in PTB or fetal loss compared with open procedures [31,32,33]. Our heatmap-based modeling, showing a steeper PTB gradient with open surgery in late gestation, is therefore consistent with a growing view that, in experienced hands and with pregnancy-specific precautions, laparoscopy is not intrinsically more hazardous and may be preferable in many scenarios.

An important and recurrent theme in our data is the apparently disproportionate obstetric risk associated with negative appendectomy, particularly in the cohort of Baruch et al. [22], where PTB was more frequent after removal of a histologically normal appendix than after inflamed appendicitis. This observation mirrors earlier concerns from a large Californian population-based analysis by McGory et al., who found that both complicated and negative appendicitis were independently associated with increased odds of fetal loss compared with simple appendicitis, and that nearly one quarter of pregnant women undergoing appendectomy had a normal appendix at histology [31]. More recently, Rottenstreich et al. reported that laparoscopic negative appendectomy during pregnancy was associated with lower gestational age at delivery and reduced neonatal birth weight compared with women with histologically confirmed appendicitis, despite similar maternal characteristics and perioperative management [32]. Taken together with our findings, these data suggest that the obstetric price paid for unnecessary surgery—through anesthesia exposure, pneumoperitoneum, postoperative inflammation, and potential iatrogenic PTB—may be at least as important as the risks conferred by appropriately treated uncomplicated appendicitis. They reinforce current diagnostic strategies that prioritize high-quality ultrasound and, where available, MRI to reduce negative appendectomy rates in pregnancy while still avoiding delay in truly inflamed cases [11,22,25,31,32].

Our synthesis also highlights the complex interplay between disease severity, timing of surgery, and PTB risk. Perforated or complex appendicitis was consistently associated with higher rates of fetal loss and preterm delivery in Zhang et al. [25] and Mantoglu et al. [26], and similar gradients have been reported in older and more recent series outside our inclusion set. Zingone et al. showed that pregnant and peripartum women with appendicitis had higher complication rates than non-pregnant counterparts, supporting the notion that delayed or missed diagnosis in pregnancy allows progression to perforation, with consequent septic and inflammatory cascades that can trigger preterm labor [30]. Multicenter data from Yoo et al. demonstrated that obstetric outcomes were largely comparable between laparoscopic and open surgery when operations were performed promptly, whereas delays were associated with more advanced pathology and worse maternal and neonatal outcomes [29]. The pattern we observed—higher PTB probability in perforated and negative appendicitis, particularly when surgery occurred in the second and third trimesters—fits with this mechanistic framework and suggests that timely, accurate diagnosis is pivotal: both overtreatment (unnecessary surgery) and undertreatment (delayed operation leading to perforation) appear to shift pregnancies toward earlier delivery.

Finally, our findings have practical implications for perioperative counseling and guideline development. Contemporary recommendations from surgical societies emphasize that laparoscopy can be safely performed in any trimester provided that intra-abdominal pressure is minimized, maternal positioning optimizes uteroplacental perfusion, and obstetric input is available, while also stressing the need to avoid non-essential procedures during pregnancy [5,18,34]. The balance of evidence from recent meta-analyses and population-based studies suggests that, for women with well-characterized appendicitis, laparoscopic appendectomy offers at least equivalent obstetric outcomes to open surgery, with advantages in recovery time and hospital resource use [21,22,23,27,28,33,34]. However, our review and the broader literature on negative appendectomy underscore that this should not translate into a lower threshold for operative exploration in equivocal cases. Instead, a pragmatic strategy may include aggressive use of imaging algorithms tailored to pregnancy, multidisciplinary decision-making, and explicit consideration of PTB risk in discussions with patients and their families. Future research should aim to integrate clinical, imaging, and biomarker data into validated risk scores that can distinguish women who would benefit most from immediate surgery from those in whom short-term observation or repeat imaging is safe, thereby minimizing both avoidable fetal exposure to surgery and the obstetric consequences of delayed treatment.

The synthesis of available data indicates that pregnant patients undergoing appendectomy face a small but clinically relevant increase in PTB risk, with crude rates approaching 10–12% in several cohorts and higher relative risks in large registry and matched-control studies. Clinicians should prioritize accurate diagnosis using contemporary imaging algorithms to reduce negative appendectomy, which appears to carry particularly high PTB risk, and should consider early involvement of multidisciplinary teams (obstetrics, surgery, anesthesia, neonatology) when surgery is required. Where expertise and gestational age permit, laparoscopic appendectomy appears at least as safe as open surgery and may be associated with lower PTB rates in some series, supporting its use with pregnancy-adapted insufflation pressures and positioning. Postoperatively, women should receive individualized counseling about heightened PTB risk, enhanced surveillance for symptoms of preterm labor, and timely access to interventions such as corticosteroids and transfer to higher-level neonatal care when indicated. Nevertheless, these findings should be interpreted in light of potential residual confounding from unmeasured or incompletely controlled factors, including underlying comorbidities and other patient- and treatment-related characteristics.

### 4.2. Study Limitations

This review is limited by the observational nature of all included studies, with inherent risks of residual confounding and selection bias. Surgical indication, intraoperative findings, and perioperative management were variably reported, and several cohorts lacked detailed adjustment for important obstetric and medical comorbidities. Heterogeneity in exposure definitions (appendicitis vs. appendectomy, inclusion of negative appendectomy), outcome reporting (crude PTB rates vs. adjusted estimates, spontaneous vs. iatrogenic PTB), and follow-up completeness precluded robust meta-analysis. Some studies were single-center with small samples, whereas others relied on administrative coding with limited clinical granularity, raising concerns about misclassification and unmeasured confounders. Temporal changes in imaging availability, laparoscopic expertise, and obstetric practice also limit the comparability of older and more contemporary datasets.

## 5. Conclusions

Appendectomy during pregnancy is consistently compatible with an elevated risk of preterm delivery, particularly when the surgery is performed as an open procedure in late gestation or results in removal of a histologically normal appendix. Nonetheless, appendectomy remains a necessary, often life-saving intervention when appendicitis is suspected, and absolute PTB risks remain moderate in most cohorts. Future research should focus on large, prospectively characterized registries that distinguish spontaneous from indicated PTB, capture detailed surgical and anesthetic variables, and better delineate the risk profile of negative appendectomy. In parallel, refinement of diagnostic pathways and perioperative obstetric care offers the best immediate strategy to minimize avoidable PTB while ensuring timely treatment of acute appendicitis in pregnancy.

## Figures and Tables

**Table 1:** Risk of Bias Assessment (Adapted from QUADAS-2).

Study	Patient Selection	Index Intervention	Confounding and Adjustments	Outcome Reporting	Overall Risk of Bias	Key Concerns
Ibiebele 2019 [21]	L	SC	L	SC	SC	Lacks surgical approach detail; raw PTB rate not provided
Baruch 2025 [22]	SC	SC	SC	L	SC	Pathology stratified well but limited surgical data
Özdemir 2025 [23]	L	L	SC	L	L/SC	Strong LA–OA design; single-center; small sample
Lindqvist 2023 [24]	SC	SC	SC	SC	SC	Registry-based; limited granularity
Zhang 2009 [25]	SC	SC	H	SC	H	Older data; incomplete pathology breakdown
Mantoglu 2020 [26]	SC	SC	SC	SC	SC	Perforation stratified; modest sample size

Abbreviations: QUADAS-2, Quality Assessment of Diagnostic Accuracy Studies 2; L, low risk of bias; SC, some concerns; H, high risk of bias.

**Table 2:** Study characteristics.

Study	Year	Country	Design	Sample Size (Pregnancies with Appendectomy)	Comparison Group	Surgical Approach Reported?	Pathology Reported?
Ibiebele et al. [21]	2019	Australia	Population registry	1024	Yes (1,124,551 pregnancies without appendicitis)	Yes (566 LA, 458 OA)	No pathology (ICD codes only)
Baruch et al. [22]	2025	Israel	Retrospective cohort	185	555 controls (3:1 matched)	Yes (117 LA, 68 OA)	Yes (141 inflamed, 44 normal)
Özdemir et al. [23]	2025	Turkey	Prospective single-center	56	None	Yes (15 LA, 41 OA)	Yes (simple vs. complex appendicitis)
Lindqvist et al. [24]	2023	Sweden	Registry-based	50	38,155 births without appendectomy	Yes (92% LA ≤ 20 w; 27% LA > 20 w)	Yes (perforation, unaffected appendix)
Zhang et al. [25]	2009	China	Hospital-based observational	78	None	NR	Yes (62 non-perforated, 16 perforated)
Mantoglu et al. [26]	2020	Turkey	Retrospective cohort	78	None	Yes (53% OA, 47% LA)	Yes (simple, suppurative, perforated, lymphoid hyperplasia)

Abbreviations: LA, laparoscopic appendectomy; OA, open appendectomy; NR, not reported; w, weeks.

**Table 3:** Maternal and surgical data.

Study	LA (n or %)	OA (n or %)	Perforated Appendicitis	Gestational Age at Surgery	Wound Infection	Other Complications
Ibiebele et al. [21]	566 (55.3%)	458 (44.7%)	NR	Median 15.4 weeks (IQR 10.4–22.3)	NR	Maternal morbidity aRR 2.68; neonatal morbidity aRR 1.42
Baruch et al. [22]	117 (63.2%)	68 (36.8%)	11/185 (5.9%)	1st: 31.9%, 2nd: 42.7%, 3rd: 25.4%	NR	Higher CS rate; 1 neonatal death
Özdemir et al. [23]	15 (26.8%)	41 (73.2%)	Complex cases reported NR exact n	Mean GA NR, includes 4–34 weeks	1 wound infection	Post-spinal headache (1 case)
Lindqvist et al. [24]	39/50 (78%) overall	11/50 (22%)	0 in second half; overall low	54% 1st tri, 36% 2nd, 10% 3rd	NR	Lower perforation rate vs. non-pregnant
Zhang et al. [25]	NR	NR	16/78 (20.5%)	All 2nd–3rd trimester	NR	Premature contractions 11%; 4 stillbirths
Mantoglu et al. [26]	37 (47.4%)	41 (53%)	9 perforated (≈11.5%)	Majority 2nd trimester	1 SSI	CRP higher in preterm/abortion group

Abbreviations: LA, laparoscopic appendectomy; OA, open appendectomy; GA, gestational age; IQR, interquartile range; aRR, adjusted risk ratio; CS, cesarean section; SSI, surgical site infection; CRP, C-reactive protein; NR, not reported.

**Table 4:** Fetal and neonatal outcomes.

Study	Preterm Birth (%)	Preterm Labor (%)	Fetal Loss (Miscarriage/IUFD)	Neonatal Death	Additional Notes
Ibiebele et al. [21]	aHR 1.73 overall; planned PTB aHR 2.08; spontaneous aHR 1.35	NR	NR	NR	Increased maternal and neonatal morbidity
Baruch et al. [22]	11.9% (vs. 5.4% controls)	NR	NA: 20.5% PTB vs. 9.2% inflamed	1 neonatal death	Negative appendix → stronger PTB signal
Özdemir et al. [23]	LA 0%, OA 24.4%	NR	0	0	LA preferred early, small sample
Lindqvist et al. [24]	4.5% (vs. 5.6% controls)	NR	1 miscarriage, 1 ectopic, 2 fetal demise pre-surgery	0	No difference in SGA or CS rates
Zhang et al. [25]	5.1% (preterm labor)	11.5% contractions	5 fetal deaths (6.4%)	1 neonatal death	Perforated appendix strongly predictive
Mantoglu et al. [26]	10.7% preterm, 5.4% abortion	NR	Abortion 5.4%	NR	Perforation → higher adverse outcomes

Abbreviations: PTB, preterm birth; LA, laparoscopic appendectomy; OA, open appendectomy; NA, negative appendectomy; IUFD, intrauterine fetal death; SGA, small for gestational age; NR, not reported.

**Table 5:** Reporting of PTB phenotype and fetal loss across included studies.

Study	PTB Subtype Reported (Spontaneous vs. Planned/Indicated)	Fetal Loss Reported (Miscarriage/IUFD/Stillbirth)	What Was Reported
Ibiebele et al. [21]	Yes	NR	Planned and spontaneous PTB analyzed separately; strongest association for planned PTB
Baruch et al. [22]	No	Limited	PTB reported; negative appendectomy subgroup higher PTB; neonatal death noted
Özdemir et al. [23]	No	Yes (0 events)	PTB 0% after laparoscopy vs. 24.4% after open; no fetal losses reported
Lindqvist et al. [24]	No	Yes	PTB reported; additional pregnancy losses described in cohort follow-up
Zhang et al. [25]	No	Yes	Perforated appendicitis associated with markedly higher fetal mortality than non-perforated
Mantoglu et al. [26]	No	Yes	Term vs. preterm vs. abortus counts provided; preterm and abortus also analyzed jointly

Abbreviations: PTB, preterm birth; IUFD, intrauterine fetal death; NR, not reported.

## Data Availability

The original contributions presented in this study are included in the article/Supplementary Material. Further inquiries can be directed to the corresponding author.

## Supplementary Materials

The following supporting information can be downloaded at: https://www.mdpi.com/article/10.3390/jcm15020819/s1, File S1: PRISMA 2020 Checklist.
